# Parental loss of family members within two years of offspring birth predicts elevated absorption scores in college

**DOI:** 10.1080/14616734.2016.1181096

**Published:** 2016-05-30

**Authors:** Naomi I. Gribneau Bahm, Robbie Duschinsky, Erik Hesse

**Affiliations:** ^a^ Department of Psychology, University of California, Berkeley, CA, USA; ^b^ Department of Public Health and Primary Care, Cambridge University, Cambridge, UK

**Keywords:** Absorption, parental loss experiences, disorganized attachment, unresolved loss, frightened/frightening parental behaviour

## Abstract

Liotti proposed that interactions during infancy with a parent suffering unresolved loss could lead to vulnerabilities to altered states of consciousness. Hesse and van IJzendoorn provided initial support for Liotti’s hypothesis, finding elevated scores on Tellegen’s Absorption Scale - a normative form of dissociation - for undergraduates reporting that their parents had experienced the loss of family members within two years of their birth. Here, we replicated the above findings in a large undergraduate sample (*N* = 927). Additionally, we investigated mother’s and father’s losses separately. Perinatal losses, including miscarriage, were also considered. Participants reporting that the mother or both parents had experienced loss within two years of their birth scored significantly higher on absorption than those reporting only perinatal, only father, or no losses. While not applicable to the assessment of individuals, the brief loss questionnaire utilized here could provide a useful addition to selected large-scale studies.

During the Adult Attachment Interview (AAI protocol; George, Kaplan, & Main, 1984, 1985, 1996), speakers are classified as Unresolved/disorganized (hereafter Unresolved) when – asked to discuss loss or abuse experiences – they exhibit brief lapses in the monitoring of speech or reasoning (AAI scoring and classification system; Main, Goldwyn, & Hesse, 2003). By 1995, a meta-analysis had indicated that infants of Unresolved parents tended to be classified as Disorganized/disoriented (hereafter Disorganized; van IJzendoorn, 1995; see also Main & Solomon, 1990 for a description of the Disorganized category) when observed with that parent in Ainsworth’s Strange Situation laboratory procedure (Ainsworth, Blehar, Waters & Wall, 1978). In three studies, Unresolved parental AAI status, as identified before the birth of the first child and thus indicative of a representational state present before any possible influence of this or another child, was found predictive of infant Disorganized attachment (mothers and infants: Fonagy, Steele, & Steele, 1991; Ward & Carlson, 1995; fathers and infants: Steele, Steele, & Fonagy, 1996). Similar results replicating the link between Unresolved and Disorganized attachment status across multiple studies have been found in a recent meta-analysis focusing largely on non-English-speaking countries (Settee, Coppola, & Cassibba, 2015). Finally, in the most recent and most comprehensive meta-analysis conducted to date (*N =* 4819; Verhage et al., 2016), Unresolved versus non-Unresolved classifications continued to yield significant effect sizes predicting infant Disorganized attachment status for both published and unpublished data (though weaker in the latter case).

The first explanation for the relation between Unresolved and Disorganized attachment status was proposed by Main and Hesse in 1990 (see also Hesse & Main, 2006). Here it was suggested that Unresolved parents might have infants vulnerable to Disorganization via parental exhibition of frightening behaviour:

threatening behaviour inexplicable in origin and/or anomalous in form: for example, in nonplay context and in the absence of metasignals of play, stiff-legged “stalking” of infant on all fours, exposure of canine tooth, hissing or deep growls directed at infant; frightened behaviour patterns inexplicable in origin and/or anomalous in form: sudden frightened look (fear mouth, exposure of whites of eyes) in absence of environmental change, also frightened retreat from the infant or approaching infant apprehensively, as though a dangerous object (Hesse & Main, 2006, p. 320).

More specifically, frightening parental behaviour was expected to present the attached infant with an irresolvable approach-flight conflict arising from the fact that the attachment figure – the infant’s biologically channelled haven of safety – had simultaneously become the source of its alarm. Further empirical work has appeared to affirm this theory (see Abrams, Rifkin, & Hesse, 2006; Jacobvitz, Leon, & Hazen, 2006; Schuengel, Bakermans-Kranenburg, & van IJzendoorn, 1999), utilizing either Main and Hesse’s (1991–1998) FR system for coding and scoring anomalous parental behaviour, or Lyons-Ruth’s AMBIANCE system (Lyons-Ruth, Bronfman, & Parsons, 1999), which includes some of the primary FR items. A meta-analysis utilizing both systems to examine the power of frightening parental behaviour to mediate the relations between Unresolved parental states of mind and infant Disorganized attachment reported substantial but incomplete mediation (Madigan et al., 2006), while a more recent study has reported full mediation (Jacobvitz, Hazen, Zaccagnino, Messina, & Beverung, 2011).

Following Main and Hesse (1990), Liotti (1992) proposed that frightening parental behaviour might lead to increased vulnerability to dissociation. This proposal was supported via Liotti’s report that, in a clinical sample, dissociative patients were significantly more likely than other psychiatric patients to report that their mothers had experienced an important loss within two years of their birth. In essence, Liotti had created a “rough and ready” estimate that the mothers (of the patients in his study) who had experienced a significant loss within this timeframe were more likely to be in an Unresolved state which, via the mechanisms described above, could under certain circumstances lead to clinical levels of dissociation in the offspring. Later, in corroboration with Liotti’s proposal, Carlson (1998) reported that Disorganization with the mother predicted dissociative phenomena during middle childhood and adolescence in the Minnesota high-risk sample (Carlson, 1998; see also Ogawa, Sroufe, Weinfeld, Carlson, & Egeland, 1997).

In 1998, Hesse and van IJzendoorn undertook a non-clinical replication of Liotti’s (1992) findings using an assessment of absorption, a “normative” form of altered consciousness. Absorption is considered a component of dissociation and is not infrequently observed in non-clinical samples. Using a large undergraduate sample, they investigated reports of parental loss of family members within two years of offspring birth, finding that participants who reported that parents had experienced the loss of a family member near the time of their birth received significantly elevated scores on Tellegen’s Absorption Scale (TAS; Tellegen & Atkinson, 1974).

Main, Hesse, and Kaplan (2005) found that infant Strange Situation security with mother, but not father, predicted participant’s adult attachment status on the AAI at age 19. Since experiencing an important loss near the birth of her child is thought to lead to an increased likelihood of the mother having been in an Unresolved state of mind with respect to attachment, thereby increasing the probability of Disorganized attachment status on the part of offspring born within this time frame (see Hesse & van IJzendoorn, 1998; Liotti, 1992), in (and only in) large-sample studies, reports of early maternal loss may be useful as a rough “estimate” of Disorganized attachment status during infancy. Two independent longitudinal studies have shown that participants Disorganized with mother in infancy are very likely to receive insecure classifications on the AAI, particularly Unresolved and Cannot Classify, as adults (Main et al., 2005; Sroufe, 2005). Furthermore, several meta-analyses have found Disorganized attachment predictive of externalizing (Cyr, Euser, Bakermans-Kranenburg, & van IJzendoorn, 2010; Fearon & Belsky, 2011) and internalizing (Groh, Roisman, van IJzendoorn, Bakermans-Kranenburg, & Fearon, 2012) disorders in later life.

In the present study, we (1) first attempt to replicate Hesse and van IJzendoorn (1998) and (2) further ask whether losses experienced specifically by mother within 24 months of participant’s birth would be especially likely to influence adolescent propensities towards absorption. To test this hypothesis, we divided the same participants’ loss categories to specifically reflect the loss history of each parent. Hence, five loss categories were delineated: mother loss only, father loss only, only perinatal loss (miscarriage/stillbirth/neonatal), both parents loss, and no loss (neither parent had a loss). Querying participants for losses by miscarriage was suggested by Hesse and van IJzendoorn, but was considered separately (in conjunction with other types of perinatal loss, e.g. stillbirth and early neonatal loss) since these types of loss were not examined in their study.

## Methods

### Participants

Study participants received course credit for research participation and consisted of 927 undergraduates^1^ enrolled in spring 2001 (*N* = 380) and spring 2002 (*N* = 547) psychology courses at the University of California, Berkeley. Students’ mean age was 20.3 years (*SD* = 3.48; range 18–55 years), and 63% were female. Students reported their ethnicities as 39.3% Asian; 26.8% Caucasian; 6.8% Latino Hispanic; 1.5% African American; and 15.6% other or mixed ethnic origin.^2^

### Measures

#### Absorption

To estimate the participant’s experiences of “episodes of ‘total attention’ that fully engage [their] representational resources” (Hesse & van IJzendoorn, 1998, p. 319), we utilized Tellegen’s Absorption Scale (Tellegen, 1982; Tellegen & Atkinson, 1974). Absorption is related to susceptibility to hypnosis (Nadon, Hoyt, Register, & Kihlstrom, 1991; Tellegen, 1982; Tellegen & Atkinson, 1974) and has been found to have a genetic component (see Finkel & McGue, 1997; Ott, Reuter, Hennig, & Vaitl, 2005; Tellegen et al., 1988). Absorption is thought to include an altered sense of self as well as an altered sense of reality in general. The TAS consists of 34 items estimating the participant’s predisposition to be absorbed by thoughts and associations. Following are five examples included in the scale:

- “While watching a show, or a play, I may become so involved that I forget about myself and my surrounding and experience the story as if it were real and as if I were taking part in it.”
- “Sometimes I ‘step outside’ my usual self and experience an entirely different state of mind.”
- “At times I feel the presence of someone who is not physically there.”
- “When listening to organ music or other powerful music, I sometimes feel as if I am being lifted into the air.”
- “I can often sense the presence of another person before I actually see or hear him/her.”

The TAS items were printed exactly as originally sequenced (Tellegen, 1982; Tellegen & Atkinson, 1974) on a two-page form embedded in a large pre-screening packet consisting of many self-report inventories being utilized in undergraduate research not related to our own investigations. The six-point Likert scale, including: −3 (strongly disagree), −2 (disagree), −1 (slightly disagree), +1 (slightly agree), +2 (agree), and +3 (strongly agree), was translated into a seven-point scale, where 4 (not available to participants) was neutral. Each participant’s score was obtained by computing the total score across the 34 items and dividing by the number of items.^3^ The absorption scale showed an alpha reliability of 0.92 (*N* = 842). The mean score for absorption was 4.55 (*SD* = 0.90; minimum 1.59, maximum 6.97; *N* = 927), and did not differ for males (4.55; *SD* = 0.88; minimum 2.44, maximum 6.74; *N* = 252) versus females (4.52; *SD* = 0.91; minimum 1.59, maximum 6.74; *N* = 588).

#### Parent’s experience of loss of family member(s) within 24 months preceding or following participant’s birth

Participants were asked whether each parent independently had suffered the loss through death of a close family member within the 24 months preceding and the 24 months following their birth. In concert with Hesse and van IJzendoorn’s (1998, p. 304) suggestion to consider “other types of losses (e.g. miscarriages)” in future studies, we requested that participants also include miscarriages and stillbirths insofar as they were aware of them. To guard against arbitrary or unconsidered responses, participants reporting parental loss experiences were asked to briefly describe the deceased person(s) and the circumstances involved.^4^ The exact wording used (with the form including two identical spaces for each parent separately) was as follows:

Did your ***mother [/father]*** lose a close family member (e.g. did her [/his] mother, father, sibling, spouse, or another child die) ***within two years*** before or after your birth? (Include stillborn children or miscarriages if you are aware of them.)

If yes, please specify the person(s) lost, the date(s) in relation to your own birth insofar as you are able, and give a brief accounting of the circumstances insofar as you are able.

My mother’s [/father’s] _____________ died approximately ______ months before/after my birth.(relation to mother [/father]) (1–24) (please circle)

Circumstances ______________________________________________________________________

For the *replication* of Hesse and van IJzendoorn’s (1998) original study,^5^ an affirmative answer to any of the questions (i.e. mother and/or father were reported to have had a loss) was considered indicative of a critical loss, and loss was treated as a dichotomous variable. Participants who reported parental loss of (exclusively) miscarriages, stillbirths or neonates were placed in a separate category, as these losses would have gone unreported in Hesse and van IJzendoorn’s study and thus needed to be studied separately. Hence, the categories of parental loss experiences examined were “Loss”, “Perinatal loss only”, and “No loss”.

For our *extension* of Hesse and van IJzendoorn’s study (1998), the loss categories were further divided to separate the loss experiences of participants’ mothers and fathers. Since mother’s loss experiences were considered likely to have a greater impact on the participant’s adult state of mind, based on longitudinal data (Main et al., 2005) linking adult attachment organization more closely to maternal than paternal Strange Situation classifications, the mother’s loss category superseded father’s when the two were in conflict. Again, maternal loss of (only) miscarriages, stillbirths or neonates was placed in a separate category, although in this analysis some of the fathers had other losses as well. (For example, if a participant reported that mother had suffered a miscarriage and father had lost his father, that participant was placed in the “Perinatal loss” category.) As such, the categories of parental loss experiences included “No loss” (75.1%), “Father loss only” (5.5%), “Perinatal loss (and father may have also experienced other losses)” (6.6%), “Mother loss only” (9.8%), and “Both loss” (3.0%). See Table 1 for a breakdown of loss categories used and the number of participants in each category.

**Table 1.** Loss categories for replication and extension.

Loss category	Replication	Extension
No loss	Neither parent had a loss, *N* = 696	Neither parent had a loss, *N* = 696
Father loss only	n/a	No loss reported for Mother; Father had a loss, *N* = 51
Perinatal loss	ONLY Perinatal loss was reported for either parent, *N* = 49 [Added to “No Loss” since not specified in original study^a^]	ONLY Perinatal loss was reported for Mother, *N* = 61 Father may have had other losses as well (*N* = 12 of 61)
Mother loss only	n/a	Mother had a loss, *N* = 91; No loss reported for Father
Loss	Either or both parents had a loss, *N* = 182	n/a
Both loss	n/a	Both Mother and Father had losses, *N* = 28
	**Total *N* = 927**	Total *N* = 927

^a^Note that removing these subjects completely would not alter statistical significance.

## Results

### Replication of Hesse and van IJzendoorn

As Hesse and van IJzendoorn (1998) had defined parental loss (i.e. either mother and/or father experiencing loss of a family member, with miscarriages not queried), 182 participants (19.6%) had a parent who had experienced a loss near the time of their birth, and the mean TAS score for these participants was 4.69 (*SD* = 0.88; minimum 2.65, maximum 6.74). These data are very similar to those reported in Hesse and van IJzendoorn (1998), where 21% of participants (*N* = 65) reported parental losses, with a mean TAS score of 4.92 (*SD* = 0.94, minimum 2.61, maximum 6.56). The mean absorption score for 745 participants whose parents had not experienced a loss was 4.52 (*SD* = 0.90; minimum 1.59, maximum 6.97), results which were again highly comparable to the earlier study (*N* = 243; mean = 4.65; *SD* = 0.93; minimum 1.29, maximum 6.74). Since miscarriages, still-births and neonatal loss were not asked for in the original study, those participants who listed only losses of this kind were included in the “no loss” category. (As noted in Table 1, removing data from these participants did not alter statistical significance.) The differences between the means in our sample was significant: *t*(925) = −2.323; *p* = .010 (one-tailed), which fully replicates Hesse and van IJzendoorn’s (1998) findings. The effect size for our sample was computed as *d* = 0.19, *r* = 0.095. Although Hesse and van IJzendoorn had not included it, we computed the effect size for their sample: *d* = 0.29, *r* = 0.143.

Sex of participants was not related to absorption. A two-way ANOVA with sex and loss as factors, and absorption as the dependent variable, showed a significant effect for loss [*F*(1,839) = 5.776, *p *= .016], with no significant interactions.

### Extension of loss categories

**Figure 1.**
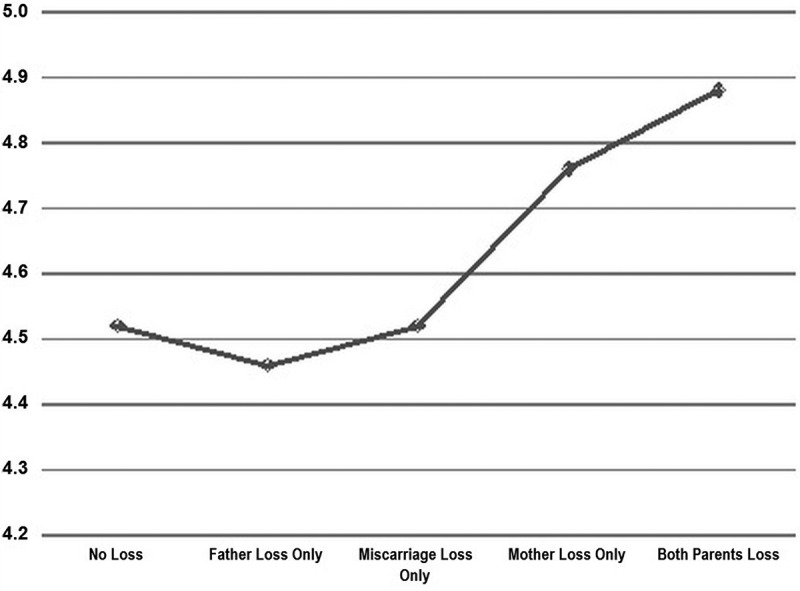
Mean TAS score by loss category.

Since longitudinal studies of attachment have suggested that the infant’s attachment relationship with its mother is the most important factor affecting adult attachment status, at least with respect to the Adult Attachment Interview (see Introduction), we expected that losses experienced by mother within 24 months of participant’s birth would have the strongest influence on adult propensities towards absorption. To test this hypothesis, we divided the participants’ loss categories to specifically reflect the mother’s (vs. father’s) loss history. Hence, five loss categories were delineated: No loss (75.1%; *N* = 696), Father loss only (5.5%; *N* = 51), Perinatal loss – including some fathers with additional losses as well (6.6%; *N* = 61), Mother loss only (9.8%; *N* = 91), and Both parents loss (3.0%; *N* = 28). A one-way ANOVA with loss as the factor and absorption as the dependent variable indicated significant differences between groups [*F*(1,926) = 2.501, *p *= .041]. See Figure 1 for a graphical depiction of the clear demarcation between these loss groups. Participants reporting that both parents experienced losses [mean 4.88; *SD* = 0.84; minimum 3.21, maximum 6.71; *N* = 28] and participants reporting that only mother experienced loss [mean 4.76; *SD* = 0.93; minimum 2.65, maximum 6.74; *N* = 91] scored higher than all other groups – participants reporting only perinatal losses [mean 4.52; *SD* = 0.87; minimum 2.26, maximum 6.47; *N* = 61], only father experienced loss [mean 4.46; *SD* = 0.76; minimum 3.00, maximum 6.03; *N* = 51], or neither parent experienced loss [mean 4.52; *SD* = 0.90; minimum 1.59, maximum 6.97; *N* = 696]. A *t*-test comparing participants whose mother or both parents had experienced a loss (mean 4.79; *SD* = 0.90; *N* = 119) to participants whose mother had experienced no losses other than miscarriages (no loss, father loss only and perinatal loss groups combined; mean 4.52; *SD* = 0.89; *N* = 808) showed that the differences were highly significant: *t*(925) = 3.058; *p* = .001 (one-tailed). The effect size was computed as *d* = 0.30, *r* = 0.149.

## Discussion

In an initial attempt to replicate and extend Liotti’s (1992) findings that dissociative patients were more likely to report that their mothers had experienced important losses within two years of their own birth, Hesse and van IJzendoorn (1998) found that measures of dissociation were highly skewed in a college sample. Hence, they utilized Tellegen’s Absorption Scale, which measures a normative form of alterations in consciousness (Tellegen, 1982; Tellegen & Atkinson, 1974) in a second sample. Here, a significant relation between reports of parental loss within this timeframe and elevated TAS responses were revealed. The current study has replicated and extended those results in a substantially larger sample.

To the extent that parents with significant losses in this two-year window are more likely than others to be Unresolved with respect to loss, they may also exhibit FR behaviours in the presence of their offspring, in turn increasing the likelihood of the offspring becoming Disorganized with respect to that parent. With this hypothesis in place, one would expect individuals reporting that both parents had experienced loss would score highest on absorption, which was in fact the case. Those who reported that mother (but not father) experienced loss were a close second. However, similar to reports of neither parent experiencing loss, reports of only father having loss experiences were not associated with higher absorption scores. Thus, absorption might become a mechanism for buffering the potentially overwhelming effects that FR behaviour specific to the mother may have upon attentional processes. While the effects of loss experiences on father alone appear to be less influential, when combined with mother having also experienced loss, we found that reports of paternal loss made a contribution to later offspring absorption. Thus, if the mother is Unresolved during the offspring’s infancy, the attachment relationship with the father may take on special import. More specifically, when the mother is Unresolved, if the father is *not* Unresolved he may become an important buffer for the infant. Conversely, if both parents are Unresolved, the infant has nowhere to turn without encountering potential FR behaviour; having two Unresolved parents is likely to be far more problematic. However, it should be noted that participants were not asked if father was present during their first two years of life, and future investigations should establish father’s presence or absence.

Elevated absorption (as measured by the TAS) has also been found associated with Unresolved status on the AAI (Hesse & van IJzendoorn, 1999) in the same individual.^6^ This finding has been replicated in a sample of 62 religious/spiritually inclined Swedish adults (Granqvist, Fransson, & Hagekull, 2009), as well as in a sample of 33 female undergraduates (Gribneau, unpublished dissertation, 2006). Gribneau also reported a significant direct correlation between these participants’ reports of mother (or both parents) having experienced a loss within two years of their birth and their Unresolved status on the AAI. Of the eight participants reporting loss experiences for mother or both parents within two years of their birth, 75% were Unresolved. For the remaining 25 participants, 10 (40%) were Unresolved. As Gribneau had hypothesized, this difference was statistically significant (*t* = 1.752; significance = 0.045, one-tailed; Gribneau, 2006, p.103). However, the correlation was no longer significant when participants who reported that *only* their father had experienced a loss within two years of their birth were included (see Gribneau, 2006, p. 116), corroborating our suggestion that the important factor predicting absorption in adulthood is the likelihood of the *mother* having been Unresolved during the participant’s infancy.

As noted in the Introduction, our extensions to Hesse and van IJzendoorn’s (1998) study included (1) their suggestion to query miscarriages (we also added stillbirth) and (2) an analysis of mother’s and father’s loss experiences separately (see above). Our findings with respect to perinatal loss were both unanticipated and somewhat puzzling. Reporting that mother had no losses other than perinatal loss (which consisted largely of miscarriages) within two years of their own birth was not associated with higher levels of absorption in our sample, whereas we expected that these losses would have had effects similar to other maternal losses. Our anticipation of this outcome had been bolstered by existing investigations which have found that maternal experiences of miscarriage and stillbirth increase both the likelihood of (1) Unresolved attachment on the part of the mother and (2) Disorganized attachment on the part of subsequent offspring (see below).

A specific query regarding miscarriage experiences was added to the loss section of the AAI by Bakermans-Kranenburg, Schuengel, and van IJzendoorn (1999), who found that 35% of their non-clinical mothers of one-year-olds reported this type of loss. The Unresolved scores the mothers’ received for their miscarriage experiences were in fact related to their infants’ Disorganized behaviours, indicating that miscarriages should be regarded as potentially important loss experiences. Unresolved loss was not related to the amount of time that had passed since the miscarriage. It is possible that mothers’ experiences of miscarriage before the birth of subsequent offspring is more influential for the infants’ outcomes than is mothers’ experiences of miscarriage after the infants’ birth (which may additionally be affected by the age of the infant at the time of the subsequent miscarriage). Separating responses of participants into “pre” and “post” birth maternal perinatal loss experiences might thus prove illuminating, with the pre-birth miscarriages being expected to predict elevated absorption tendencies.

Next-born children after a mother’s experience of stillbirth have been found significantly more likely to be Disorganized (Hughes, Turton, Hopper, McGauley, & Fonagy, 2001), which was not accounted for by differences in demographics or maternal psychiatric symptoms. Instead, Disorganization was strongly predicted by the mother’s Unresolved status on the AAI. The authors concluded that “the strong association between disorganisation of infant attachment and maternal state of mind with respect to loss suggests that the mother’s state of mind may be causal”. These same researchers later reported (Hughes, Turton, Hopper, McGauley, & Fonagy, 2004) that while women who had suffered stillbirth were more likely to be Unresolved than control women in general, higher Unresolved scores (in relation to stillbirth) were associated with experiences of childhood trauma, poor family support after the loss, and having a funeral for the stillborn infant.

Thus, it is possible that the effects of perinatal loss are highly diverse, leading to greater lability in the degree of adversity of outcome. Additionally, although miscarriages are no doubt ordinarily stressful events, they may not be as commonly experienced with the same intensity as other losses. It is also probable that participants reporting on parental losses within two years of their birth are less likely to have been informed of miscarriages. This could skew responses and consequently restrict the ability to detect full impact. We had presumed that if a participant knew about a miscarriage occurring within two years of their birth, it would have been more likely to have been of special import to the parent(s), which – at least in terms of correlating with higher absorption scores for the participant – was not supported in our sample. We would therefore suggest that future investigations may benefit from collecting more detailed information regarding the circumstances surrounding any perinatal loss experiences reported (e.g. whether the loss was the first pregnancy, whether it occurred before or after participant’s birth, whether other potential risk factors for the mother were present, etc.).

## Conclusions and future directions

In this study, we were able to fully replicate Hesse and van IJzendoorn’s (1998) finding of elevated absorption scores in adolescence for individuals born within a 24 month period of reported parents’ important loss experiences. However, despite our substantially larger sample, the mean differences in absorption scores between the groups remained no greater than in the previous study, suggesting that either the relation between the phenomena is inherently relatively small, or that our measures as developed to this point in time were not able to more fully isolate it.

With respect to the modest effect sizes found both in this study and the original investigation by Hesse and van IJzendoorn (1998), we offer several possible explanations which could contribute individually or in combination. One obvious problem is that, like miscarriages, not all parental losses may be known to offspring. With respect to losses that are reported, it is likely that:

1. many parents experiencing losses within this time period do not subsequently become Unresolved. Some salient buffering factors could include being in a secure state of mind with respect to attachment and/or being in an otherwise ecologically supportive context (e.g. supportive partner, friends and/or family members);
2. in the event that a parent *does* become Unresolved with respect to a recently experienced loss, they may not ultimately display FR behaviour;
3. not all infants exposed to FR behaviour become Disorganized (for a discussion on gene–environment interactions and Disorganized attachment, see Bakermans-Kranenburg & van IJzendoorn, 2007); and
4. not all Disorganized infants will exhibit propensities towards dissociation or absorption.

Additionally, there are likely to be other as yet unspecified pathways to propensities for absorption. Taking all these factors into consideration, we should not expect strong links between a retrospective report of parental loss within two years of a participant’s birth and levels of absorption in adulthood.

Finally, we further extended earlier findings by dividing the reported parental loss experiences into more fine grained categories. Through these analyses, it became apparent that the mother’s loss experiences were the most significant factor related to elevated absorption scores in offspring. Father’s loss experiences made an important contribution only when mother also experienced loss: the combination of both parents having experienced loss was, in fact, associated with the highest mean absorption scores.

Further investigation of this topic would of course ideally involve a prospective longitudinal study which incorporated soliciting information regarding parental loss experiences surrounding offspring birth. Alternatively, parents of school-aged children could be queried regarding their loss experiences around the time of the child’s birth (which is likely to be far more accurate than offspring’s retrospective reports) and then children with and without parental loss experiences in early years could be subsequently given measures of absorption, dissociation, et alia, in later years. Parents could also be queried regarding the circumstances of any losses reported and ecological factors such as level of perceived emotional support and marital satisfaction. This type of design would allow for moderation and mediation analyses, which might more incisively elucidate the relation between absorption and parental loss experiences. Other studies should aim to differentiate parental loss experiences from additional potential stressors which might produce subsequent offspring absorption. Potential correlates of absorption (e.g. concurrent stress, depression/other clinical symptoms) in the participants themselves should also be explored.

Reports of parental loss experiences as utilized in this study are, of course, only a rough estimate for the likelihood of concomitant Unresolved states of mind on the AAI, and accordingly increased infant Disorganized attachment for the offspring. As Disorganized attachment is a known risk factor for psychopathology (see Introduction), and there is an increasing awareness of relations between emotional and physical well-being (Reblin & Uchino, 2008), queries regarding parental loss within a 24 month period before or after an individual’s birth could potentially inform large-scale epidemiological studies exploring not only psychological but physical health outcomes as well.

## Notes

1. TAS inventories were collected from 970 participants. However, 13 of these participants completed the TAS twice, either from the same semester (N = 3) or across the two semesters (N = 10). Insofar as possible, each student’s first response was retained. For the remaining 944 unique participants, three had missing pages, eight had circled more than one response to the same TAS item, and 19 had left more than one TAS item blank. These inventories were determined to be unreliable and were discarded, resulting in a final sample size of 927.

2. Due to the nature of data collection and entry, each demographic category had missing values as follows: Age: N = 83, 9.0%; gender: N = 87, 9.4%; and ethnicity: N = 93, 10.0%.

3. For the 84 participants who skipped one TAS item, the total score of items answered was divided by 33.

4. If the participant listed only parental losses that were outside the ± 24 month window (N = 6), they were considered to have reported no loss experiences. Also, abortions (N = 3) were not counted as loss experiences for the purpose of this research.

5. Specifically “Study 2”. (Study 1 queried parental loss of participant’s siblings only.)

6. Thomson and Jaque (2012) have additionally reported correlations between greater propensities towards both dissociation and supernatural beliefs in college students and community members with Unresolved AAI classifications.
